# Internal blood lead exposure levels in permanent residents of Jiangxi Province and its effects on routine hematological and biochemical indices

**DOI:** 10.3389/fpubh.2024.1357588

**Published:** 2024-09-09

**Authors:** Wenxin He, Junjie Fu, Ruiyi Fu, Xiaoguang Song, Siyue Huang, Yujue Wang, Keke Lu, Hao Wu

**Affiliations:** ^1^Jiangxi Provincial Center for Disease Control and Prevention, Nanchang, Jiangxi, China; ^2^School of Public Health, Nanchang University, Nanchang, Jiangxi, China; ^3^Faculty of Business and Economics, The University of Melbourne, Parkville, VIC, Australia; ^4^Dermatology Hospital of Jiangxi Province, Nanchang, Jiangxi, China; ^5^Nanchang Health Promotion Center, Nanchang, Jiangxi, China; ^6^Jiangxi Provincial Patriotic Hygiene and Health Promotion Center, Nanchang, Jiangxi, China

**Keywords:** blood lead levels, hematological indices, biochemical indices, heavy metals, biomonitoring

## Abstract

**Background:**

Lead exposure levels are closely linked to human health and can cause damage to multiple organ systems, including the blood system and liver. However, due to insufficient evidence, the effects of lead exposure on hematological and biochemical indices have not been fully established.

**Objective:**

This study aims to explore the blood lead levels of permanent residents in Jiangxi Province and analyze the factors affecting blood lead levels and the impact of blood lead levels on hematological and biochemical indices.

**Methods:**

We conducted a cross-sectional study including questionnaires, health examinations, and blood sample examinations on 720 randomly selected permanent residents (3–79 years) in Jiangxi Province in 2018. The blood lead levels were measured using inductively coupled plasma mass spectrometry. Routine hematological and biochemical tests were determined by qualified medical institutions using automated hematology analyzers and biochemistry analyzers.

**Results:**

The geometric mean of blood lead concentration in permanent residents of Jiangxi Province was 20.45 μg/L. Gender, age, annual household income, smoking, and hypertension were the influencing factors for blood lead levels. For each 1 μg/L increase in blood lead, the risks of elevated red blood cell count (from low to high), platelet volume distribution width, alkaline phosphatase (from low to high), and cholesterol increased by 2.4, 1.6, 3.6, and 2.3%, respectively, whereas the risks of elevation of direct bilirubin and total bilirubin both decreased by 1.7%.

**Conclusion:**

The blood lead level in permanent residents of Jiangxi Province is higher than the national average. Higher blood lead levels were found in men than in women; blood lead levels were positively correlated with age but negatively correlated with annual household income; smoking and hypertension are risk factors for elevated blood lead; and blood lead levels affect routine hematological and biochemical markers such as red blood cell count, platelet volume distribution width, direct bilirubin, total bilirubin, alkaline phosphatase, and cholesterol.

## 1 Introduction

Lead, also known as Pb, is a toxic heavy metal element that is widely found in human production and the living environment. Humans are exposed to Pb mainly through their diet, drinking water, air, and industrial production such as heavy metals, fuel, batteries, and gasoline manufacturing (1). In terms of diet, the test results of a total of 1,065 food items in six categories in Jiangxi Province during the period from 2018 to 2021 showed that the exceedance rate of lead was 0.75% (2). In terms of soil and water, some river water bodies in Jiangxi Province have high levels of lead (3–5); the lead content in river sediments is generally higher than the soil background value (3, 6, 7); and the lead content of Jiangxi soils, especially those in mining areas, is serious soil background value (8–12). In terms of air, atmospheric lead emissions in Jiangxi Province have increased in recent years, from 23rd in the country in 2002 (less than 200 tons) to 11th in the country in 2017 (nearly 400 tons), with non-ferrous metal smelting, industrial coal combustion, and iron and steel smelting being the main sources of lead (13). In terms of agriculture and industry, there have been reports indicating that workers in certain industries in Jiangxi Province have seriously exceeded blood lead levels (14, 15), and incidents of lead contamination, such as from improperly treated sewage from storage batteries (16) and lead contamination due to irrigation of swine wastewater (17), have also occurred occasionally. In summary, there is a certain risk of environmental lead exposure in Jiangxi Province.

Blood is both a transport medium and a key toxic target for lead. Once in circulation, blood lead levels reflect the balance of tissue absorption and accumulation. According to the clinical guidelines for blood lead levels of the China Health Council, 400 μg/L is considered harmful to health for adults and 100 μg/L for children. However, none of the blood lead values are considered safe. Due to the high toxicity and slow clearance rate, even small amounts of lead can cause damage to the nervous system, cardiovascular system, reproductive system, hematopoietic system, and liver health (18–20).

Lead blood exposure has been found to have significant effects and alterations on many hematological indicators (21). Most studies consider Pb as a harmful hematological factor that can lead to a decrease in red blood cell count, mean platelet volume, and hemoglobin content. However, some studies have come to the opposite conclusion (22, 23), Chwalba et al. (24) suggested that long-term lead exposure at levels of <50 μg/dL did not affect red blood cell counts and hemoglobin levels, and that long-term lead exposure elevated mean platelet volume compared to short-term exposure, suggesting that the relationship between lead exposure and hematological parameters is unclear and that different levels of lead exposure may have different effects on hematological indices The liver is both an important detoxification organ in the body and the site of initial lead storage and damage. Lead absorbed into the circulation reaches the liver rapidly, where it is metabolized, accumulated, and excreted through the liver, causing pathological and biochemical alterations in these organs and thus indirectly affecting blood biochemical parameters. Some studies have shown that blood lead is positively correlated with total cholesterol (25), and liver enzymes (ALP, ALT, and AST) while negatively correlated with direct bilirubin (26). However, an animal study by Pandi Prabha showed that lead toxicity reduced the levels of the liver marker enzymes alanine aminotransferase and aspartate aminotransferase in fish serum (27).

In conclusion, it is important to pay attention to the lead exposure issue in Jiangxi Province. However, the current research on blood lead and human health indicators focuses on high-risk groups or high levels of lead exposure, and there are not many human studies on the monitoring of lead exposure in the general population and the impact of low-level blood lead exposure on blood and biochemical indicators, and the relevant studies in Jiangxi Province are still blank. The connections among blood lead and hematological and biochemical indices are not conclusive yet. Therefore, we investigated the status of lead blood internal exposure in permanent residents of Jiangxi Province, aiming to understand the blood lead levels and their effects on routine hematological and biochemical indices in permanent residents of the province, to provide research evidence to elaborate the mechanisms of lead effects on routine hematological and biochemical indicators, and to provide a scientific basis for the development of targeted health strategies.

## 2 Materials and methods

### 2.1 Sample size estimation

The minimum sample size of the monitoring sites was determined using the formula sample size:

$$n={\left(\frac{z_{\alpha ∕2}S}{\varepsilon \overset{-}{X}}\right)}^{2}\mathrm{deff}$$

Where *α* = 0.05, the degree of certainty z*_α/2_* was 1.96, the relative error ε was taken as 10%, *^−^X* and *S* was taken as the mean and standard deviation of environmental lead exposure in China, 24.98 and 10.75, respectively (28), and the design effect *deff* was estimated as 2. The minimum sample size *n* was calculated to be 142. Taking into account the feasibility of the project, national and local financial support, and the balanced distribution of gender and age, it was determined that the monitoring would be carried out in five monitoring sites across the province based on meeting the sampling requirements, with 144 people in each monitoring site for a total sample size of 720 people.

### 2.2 Study population

The study population was permanent residents aged 3–79 years in the survey area (living in the monitoring area for more than 6 months in the 12 months prior to the survey). Permanent residents for the purpose of this monitoring were defined as citizens of Chinese nationality who had lived in the monitoring area for more than 6 months in the 12 months prior to the survey, excluding residents in the functional areas of their residences, such as the military, schools, nursing homes, and so on.

### 2.3 Sampling methods

The method of multi-stage stratified random sampling was adopted, based on the urbanization rate and secondary industry employment rate of 100 counties (districts and cities) in Jiangxi Province, after stratification by clustering method, systematic sampling was used to select five monitoring sites in Duchang County, Jizhou District, Jinxian County, Lushan City and Qingshanhu District. Each monitoring site was divided into two strata according to urban and rural areas, and the urban/rural sampling ratio for the three survey sites was determined on the basis of the urbanization rate. Based on the demographic information obtained from the survey sites, the population was divided into 6 strata according to age: 3–5, 6–11, 12–18, 19–39, 40–59, and 60–79 years old, and within each stratum, it was further divided into 2 strata according to gender, for a total of 12 strata, with 4 randomly selected samples in each stratum, for a total of 720 permanent residents.

### 2.4 Questionnaires and health checks

Participants were interviewed in person using face-to-face questioning by uniformly trained investigators, and younger children were answered by their parents on their behalf. The survey included basic information such as gender, age, place of origin, transportation travel, diet and home environment, as well as daily behavioral habits such as smoking and drinking. Smoking and drinking status were defined as current cigarette consumption and alcohol consumption within 1 year, and any subject under 10 years of age was considered to be a non-smoker and non-drinker.

The health examination includes height, weight, hematology routine, and blood biochemistry items. Height measurement was performed using a metal column type height meter with an accuracy of 0.1 cm; weight measurement was performed using an electronic weight scale with an accuracy of 0.1 kg The body mass index (BMI) of subjects aged 3 ~ 6 years, 7 ~ 17 years and 18 years and above were categorized as wasting, normal, overweight, and obesity according to the appropriate criteria (29–32). The clinical hematology routine and biochemical tests were determined by qualified medical institutions using automated hematology analyzers and biochemistry analyzers. The routine blood tests refer to WS/T 406–2012 Clinical Hematology Testing Routine Items Analysis Quality Requirements, and the biochemical tests refer to WS/T 403–2012 Clinical Biochemistry Testing Routine Items Analysis Quality Indicators. The test results were uploaded to the information management platform system of the center in Excel format.

### 2.5 Sample collection and processing

Sample collection and processing were carried out in strict compliance with the Biological Monitoring Quality Assurance Specification (GB/T 16126) (33). Blood samples were collected using 5 mL vacuum blood collection tubes, 3 tubes of fasting venous blood non-anticoagulated whole blood, and 1 tube of anticoagulated whole blood of 4 mL each for children over 12 years old and adults; 1 tube of fasting venous blood anticoagulated whole blood and 2 tubes of non-anticoagulated whole blood of 4 mL each for children 6–12 years old; and 1 tube of 4–5 mL anticoagulated blood for children under 6 years old.

### 2.6 Sample transport, preservation, and testing

All samples were dispensed within 4 h. Samples for hematological tests and blood biochemical tests could only be stored at 2 ~ 6°C within 24 h of dispensing and were not to be frozen. Samples for blood lead tests were transported to the Chinese Center for Disease Control and Prevention within 24 h of dispensing for detection by inductively coupled plasma mass spectrometry (ICP-MS). The detection limit (LOD) for blood lead was 0.035 μg/L, and all test values were above the detection limit.

### 2.7 Quality control

Organize unified training for investigators before conducting the survey, standardize the questionnaire survey process and filling methods, and arrange for supervisors to supervise and guide the survey site to ensure that the survey is conducted in strict accordance with the requirements of the unified workbook. The collection of blood samples from survey subjects is undertaken by qualified medical institutions; sampling supplies are uniformly issued; each batch of samples is measured in a standard series; the linear correlation coefficient of the standard curve for lead elements should be ≥0.999; at least 3 sets of field blanks are prepared for each batch of samples; and the sample blanks are not higher than the detection limit. Health checkups were conducted in qualified medical institutions, and the testing instruments all met the requirements of national metrological certification. The data were entered into the computer system and reviewed by Jiangxi CDC and China CDC to ensure the reliability of the data.

### 2.8 Statistical methods

Statistical analysis using SPSS (version 26.0). The blood lead levels of the study subjects were skewed and approximately obeyed normal distribution after logarithmic transformation, described by geometric mean (G), median and interquartile range [*M* (P_25_ ~ P_75_)], maximum and minimum values. Combining the questionnaire and health examination data, the Mann–Whitney rank sum test was used for the comparison of two independent samples, the Kruskal-Wallis rank sum test was used for the comparison of multiple independent samples, and Spearman’s correlation was used to determine the correlation between the two skewed distribution indicators. Variables for which the rank-sum test or Spearman’s correlation was statistically significant were included in the linear regression. The data on routine hematological and biochemical indexes were skewed and classified into low (< P_25_), medium (P_25_ ~ P_75_), and high (> P_75_) levels according to the range of P_25_ ~ P_75_. After correcting for potential confounders, logistic regression was used to explore the dose–response relationship between blood lead levels and routine hematological and biochemical parameters. A *p* < 0.05 was considered a statistically significant difference.

## 3 Results

### 3.1 Demographic characteristics of the study population

The demographic characteristics of the study population are shown in Table 1. There were 720 study subjects, of which 360 (50.00%) were male and 360 (50.00%) were female; 384 (53.33%) were urban residents and 336 (46.67%) were rural residents; the age of the study subjects (29.29 ± 23.21) years, range 3 ~ 78 years, 3 ~ 5, 6 ~ 11, 12 ~ 18, 19 ~ 39, 40 ~ 59, 60 ~ 79 years old were 121 (16.81%), 119 (16.53%), 119 (16.53%), 120 (16.67%), 121 (16.81%), 120 (16.67%), respectively. 47 (6.53%), 427 (59.30%), 183 (25.42%), and 63 (8.75%) of the study subjects were wasting, normal, overweight, and obesity, respectively. Annual household income was less than 30 thousand yuan, 30 ~ 100 thousand yuan, and more than 100 thousand yuan were 168 (23.33%), 369 (51.25%), and 146 (20.28%). Seventy (9.72%), 49 (6.81%), 167 (23.19%), and 434 (60.28%) of the study population were engaged in primary, secondary, tertiary and other occupations, respectively. Nine (1.25%) of the study population had occupational pollution from metals and metalloids, 31 (4.31%) had occupational pollution from pesticides, 36 (5.00%) had occupational pollution from production dusts, and 37 (5.14%) had other occupational pollution.105 (14.58%), 185 (25.69%), and 44 (6.11%) were smokers, alcohol drinkers, and hypertensive patients, respectively (Table 1).

**Table 1:** Univariate analysis of blood lead exposure level (μg/L) and demographic characteristics, smoking, alcohol consumption, and hypertension in permanent residents of Jiangxi Province.

Features	Number of cases (composition ratio/%)	G	*M* (_P25 ~ P75_)	Statistical values	*p*
Blood lead	720 (100.00)	20.45	20.03 (15.12 ~ 26.54)		
Gender					
Male	360 (50.00)	22.84	22.00 (17.21 ~ 29.75)	−7.48^a^	< 0.001
Female	360 (50.00)	18.30	17.20 (13.69 ~ 23.21)		
Age group (years)					
3 ~ 5	121 (16.81)	19.44	19.09 (15.43 ~ 24.38)	117.06^b^	< 0.001
6 ~ 11	119 (16.53)	18.34	18.13 (13.69 ~ 23.25)		
12 ~ 18	119 (16.53)	16.71	16.78 (13.52 ~ 20.80)		
19 ~ 39	120 (16.67)	18.18	17.21 (14.09 ~ 23.74)		
40 ~ 59	121 (16.81)	25.01	24.57 (17.94 ~ 32.3)		
60 ~ 79	120 (16.67)	26.88	25.76 (20.11 ~ 36.53)		
Fat and thinness					
Wasting	49 (6.81)	19.55	20.68 (13.65 ~ 24.6)	13.61^b^	0.003
Normal	420 (58.33)	19.82	19.02 (14.92 ~ 25.02)		
Overweight	188 (26.11)	22.35	21.70 (16.52 ~ 29.65)		
Obesity	63 (8.75)	20.03	18.40 (13.98 ~ 28.87)		
Urban and rural					
Urban	384 (53.33)	20.10	18.97 (14.92 ~ 25.81)	−1.69^a^	0.091
Rural	336 (46.67)	20.86	20.81 (15.69 ~ 26.84)		
Annual household Income (thousand yuan)					
<370	168 (23.33)	22.19	21.79 (16.42 ~ 29.06)	16.80^b^	0.001
30–100	369 (51.25)	20.62	20.08 (15.52 ~ 26.92)		
>100	146 (20.28)	18.63	17.41 (14.34 ~ 23.84)		
Unknown	37 (5.14)	18.77	17.77 (13.65 ~ 24.81)		
Occupation**					
Primary industry	70 (9.72)	24.81	24.05 (17.96 ~ 35.16)	60.44^b^	< 0.001
Secondary sector	49 (6.81)	27.37	26.67 (22.42 ~ 33.89)		
Tertiary	167 (23.19)	21.62	20.62 (15.65 ~ 29.67)		
Other occupations	434 (60.28)	18.77	18.08 (14.61 ~ 23.6)		
Occupational pollution					
No	607 (84.31)	19.99	19.2 (14.96 ~ 25.73)	17.56^b^	0.002
Metals and Metalloids	9 (1.25)	30.14	27.31 (23.06 ~ 40.05)		
Pesticides	31 (4.31)	22.48	21.39 (17.77 ~ 28.58)		
Production Dust	36 (5)	24.07	23.66 (18.40 ~ 29.65)		
Other Pollution	37 (5.14)	21.33	21.82 (14.16 ~ 29.99)		
Smoking					
Yes	105 (14.58)	28.37	28.28 (21.85 ~ 36.57)	−8.46^a^	< 0.001
No	615 (85.42)	19.34	18.79 (14.76 ~ 24.54)		
Drinking					
Yes	185 (25.69)	24.12	23.70 (17.28 ~ 32.27)	−5.93^a^	< 0.001
No	535 (74.31)	19.31	18.64 (14.81 ~ 24.57)		
Hypertension					
Yes	44 (6.11)	28.08	29.42 (20.19 ~ 36.89)	−24.21^a^	< 0.001
No	676 (93.89)	20.03	19.47 (14.95 ~ 25.47)		

**The primary sector refers to agriculture, forestry, animal husbandry and fisheries. The secondary sector refers to mining, manufacturing, electricity, heat, gas and water production and supply, and construction. The tertiary industry includes: information transmission, software and information technology services, finance, wholesale and retail trade, education, health and social work, transportation, warehousing and postal services, accommodation and catering, real estate, international organizations, leasing and business services, culture, scientific research, social security and social organizations, water conservancy, environment and public facilities management, and repair and recreation. ^a^Statistics are Z-values; ^b^Statistics are H-values.

### 3.2 Transportation travel, diet, and home environment of the study population

The study subjects traveled to and from work or school mainly on foot, 250 (34.72%) of them, and the travel time to and from work or school was mainly within one hour. Most of the study subjects made breakfast, lunch and dinner at home, 561 (77.92%), 659 (91.53%) and 477 (66.25%) respectively. The median and quartiles of mean daily intake of beverages, boiled water, coffee, freshly squeezed juice, bottled water, raw water, tea, staple food, meat, fish intake, eggs, vegetables, fruits, milk, mushrooms, and other food items of the study population in the past year were, respectively, 0 (0 ~ 20) mL/d, 600 (400 ~ 800) mL/d, 0 (0 ~ 0) mL/d, 0 (0 ~ 0) mL/d, 0.83 (0,57 ~ 14) mL/d, 0 (0 ~ 0) mL/d, 0 (0 ~ 0) mL/d, 346.76 (264.07 ~ 487.99) g/d, 60.14 (27.72 ~ 113.83) g/d, 14.29 (4.12 ~ 35.12) g/d, 30 (14.29 ~ 50) g/d, 155 (80 ~ 300) g/d, 50 (14.29 ~ 120) g/d, 35.71 (0 ~ 200) g/d, 3.57 (1 ~ 14.29) g/d, 8.09 (1.67 ~ 23.33) g/d. Among the study subjects, 539 (74.86%) had tap water as their main type of drinking water, 652 (90.56%) had a frequency of consumption of fried foods <1 time/week, and 673 (93.47%) had a frequency of consumption of barbecued foods <1 time/week (Tables 1, 2).

**Table 2:** Univariate analysis of blood lead exposure level (μg/L) and transportation and diet among permanent residents in Jiangxi Province.

Features	Number of cases (composition ratio/%)	G	*M* (P_25 ~ P75_)	Statistical values	*p*
Mode of travel to and from work or school					
No travel	191 (26.53)	22.85	22 (15.99 ~ 31.97)	25.10^b^	0.001
Walking	250 (34.72)	18.78	18.12 (14.76 ~ 22.98)		
Bicycle	33 (4.58)	19.77	19 (16.1 ~ 25.39)		
Electric scooter/motorcycle	156 (21.67)	20.39	20.35 (14.82 ~ 26.94)		
Car	54 (7.5)	21.2	20.62 (15.83 ~ 26.63)		
Public Transportation	27 (3.75)	18.62	17.67 (14.76 ~ 24.17)		
Subway	3 (0.42)	21.56	20.21 (13.33 ~ 20.21)		
Other	6 (0.83)	28.74	28.26 (19.62 ~ 43.05)		
Travel time to and from work or school					
0	192 (26.67)	22.96	22.4 (16.02 ~ 32.08)	16.12^b^	0.001
≤1 (h/d)	394 (54.72)	19.72	18.98 (14.86 ~ 24.6)		
1 ~ 2 (h/d)	92 (12.78)	19.43	19.34 (15.84 ~ 25.11)		
>2 (h/d)	42 (5.83)	18.91	18.61 (14.41 ~ 25.31)		
Main dining place for breakfast					
Made at home	561 (77.92)	20.45	19.91 (15.18 ~ 26.69)	0.67^b^	0.880
Bought at home	25 (3.47)	21.35	21.8 (15.68 ~ 26.42)		
Restaurant	66 (9.17)	20.71	19.61 (15.38 ~ 25.82)		
School	68 (9.44)	19.90	20.2 (14.66 ~ 25.98)		
Main place for lunch					
Make at home	659 (91.53)	20.52	19.97 (15.24 ~ 26.46)	0.52^b^	0.915
Buy at home	3 (0.42)	19.08	20.51 (14.78 ~ 20.51)		
Restaurants	12 (1.67)	18.89	16.93 (14.81 ~ 26.38)		
Schools	46 (6.39)	19.97	20.94 (13.08 ~ 29.5)		
Main place for dinner					
Make at home	477 (66.25)	20.86	20.21 (15.24 ~ 27.26)	3.40^b^	0.334
Buy at home	6 (0.83)	24.12	23.53 (15.81 ~ 36.05)		
Restaurant	21 (2.92)	19.1	16.29 (14.19 ~ 24.98)		
School	216 (30)	19.61	19.63 (14.92 ~ 24.7)		
Beverage intake (mL/d)			0 (0 ~ 20)	−0.024^c^	0.525
Boiled water intake (mL/d)			600 (400 ~ 800)	0.052^c^	0.161
Coffee intake (mL/d)			0 (0 ~ 0)	−0.016^c^	0.677
Freshly squeezed fruit juice intake (mL/d)			0 (0 ~ 0)	−0.042^c^	0.262
Bottled water intake (mL/d)			0.83 (0 ~ 57.14)	−0.039^c^	0.296
Raw water intake (mL/d)			0 (0 ~ 0)	0.031^c^	0.413
Tea intake (mL/d)			0 (0 ~ 0)	0.163^c^	< 0.001
Staple food intake (g/d)			346.76 (264.07 ~ 487.99)	−0.009^c^	0.807
Total meat intake (g/d)			60.14 (27.72 ~ 113.83)	0.027^c^	0.464
Fish intake (g/d)			14.29 (4.12 ~ 35.12)	−0.006^c^	0.882
Total egg intake (g/d)			30 (14.29 ~ 50)	−0.047^c^	0.204
Vegetable intake (g/d)			155 (80 ~ 300)	−0.019^c^	0.606
Fruit intake (g/d)			50 (14.29 ~ 120)	−0.042^c^	0.259
Milk intake (g/d)			35.71 (0 ~ 200)	−0.070^c^	0.059
Intake of mushrooms (g/d)			3.57 (1 ~ 14.29)	0.029^c^	0.433
Other food intake (g/d)			8.09 (1.67 ~ 23.33)	−0.002^c^	0.958
Drinking water type					
Tap water	539 (74.86)	20.28	19.48 (15.05 ~ 26.46)	2.46^b^	0.482
Bucket/bottle water	37 (5.14)	19.79	20.8 (14.1 ~ 23.91)		
Well water	137 (19.03)	21.28	20.92 (16.13 ~ 27.77)		
Others	7 (0.97)	20.86	18.95 (15.99 ~ 28.45)		
Frequency of consumption of fried food					
< 1 time/week	652 (90.56)	20.54	19.99 (15.22 ~ 26.72)	−0.72^a^	0.470
≥ 1 time/week	68 (9.44)	19.56	20.18 (14.4 ~ 24.47)		
Barbecue food consumption frequency					
< 1 time/week	673 (93.47)	20.58	20.08 (15.2 ~ 26.72)	−1.26^a^	0.206
≥ 1 time/week	47 (6.53)	18.69	18.32 (13.69 ~ 24.29)		

^aStatistics are Z-values; bstatistics are H-values; cstatistics are rs.^

Among the study subjects, 52 (7.22%) used air purifiers or activated charcoal. Most of the study subjects did not use insecticides, moth-proofing agents, air fresheners, air purifiers, disinfectants, toilet cleaners, and hoods on a regular basis, 451 (62.64), 598 (83.06%), 661 (91.81%), 698 (96.94%), 527 (73.19%), 286 (39.72%), and 326 (45.28%). 352 (48.89%) used mosquito repellent occasionally. Among the study population, 365 (50.69%) did not renovate their dwellings, 442 (61.39%) did not replace their furniture, 349 (48.47%) had simple buildings, 319 (44.31%) had actual usable area of the dwelling less than 100 m2, 473 (65.69%) used closed kitchens, 384 (53.33%) had kitchens with exhaust fan for ventilation, 595 people (82.64%) use gas/LPG/natural gas/biogas as the first domestic fuel for cooking, 518 people (71.94%) cook frequently, 516 people (71.67%) use electricity as the main heating method in winter, 653 people (90.69%) in spring, 686 people (95.28%) in summer, and 627 people (87.08%) in fall, 455 (63.19%) in winter, and the frequency of indoor ventilation was characterized by >5 times/week.

### 3.3 Blood lead internal exposure levels and univariate analysis

The blood lead concentration of 720 study subjects participating in this survey ranged from 7.27 to 103.73 μg/L, with a geometric mean of 20.45 μg/L, a median of 20.03 μg/L, and P_25_ and P_75_ were 15.12 and 26.54 μ g/L, respectively. The results of the rank sum test showed that blood lead levels differed significantly by gender (*p <* 0.001), age group (*p <* 0.001), fat and thinness (*p =* 0.003), annual household income (*p =* 0.001), occupation (*p* < 0.001), occupational pollution (*p* < 0.05), smoking (*p <* 0.001), drinking (*p <* 0.001), hypertension (*p <* 0.001), mode of travel to and from work or school (*p* < 0.05), travel time to and from work or school (*p* < 0.05), frequency of hood use (*p* < 0.05), type of housing (*p* < 0.05), and frequency of cooking (*p* < 0.05). Blood lead levels were positively correlated with tea intake (*p* < 0.001) (Tables 1, 2, 3).

**Table 3:** Univariate analysis of blood lead exposure level (μg/L) and home environment in permanent residents of Jiangxi Province.

Features	Number of cases (composition ratio/%)	G	M (_P25 ~ P75_)	Statistical values	*p*
Whether to use air purifier or activated carbon					
No	668 (92.78)	20.49	19.99 (15.22 ~ 26.54)	−0.52^a^	0.601
Yes	52 (7.22)	19.88	20.48 (14.02 ~ 26.6)		
Frequency of insecticide use^d^					
No	451 (62.64)	20.26	19.58 (14.83 ~ 26.67)	2.60^b^	0.457
Occasionally	208 (28.89)	20.84	20.34 (15.86 ~ 26.6)		
Sometimes	55 (7.64)	20.15	19.67 (14.83 ~ 24.11)		
Often	6 (0.83)	24.52	25.91 (18.64 ~ 35.35)		
Frequency of mosquito repellent use^d^					
None	152 (21.11)	20.72	19 (15.29 ~ 27.09)	1.80^b^	0.616
Occasionally	352 (48.89)	20.12	19.44 (14.88 ~ 26.1)		
Sometimes	190 (26.39)	20.87	21.02 (15.25 ~ 27.12)		
Often	26 (3.61)	20.31	20.39 (15.59 ~ 23.66)		
Frequency of use of moth-proofing agents^d^					
No	598 (83.06)	20.42	20.15 (15.16 ~ 26.68)	4.37^b^	0.225
Occasionally	73 (10.14)	19.76	18.32 (14.79 ~ 25.07)		
Sometimes	20 (2.78)	19.34	19.41 (14.01 ~ 26.43)		
Often	29 (4.03)	23.71	22.28 (17.06 ~ 33.78)		
Frequency of air freshener use^d^					
None	661 (91.81)	20.45	19.97 (15.1 ~ 26.45)	0.49^b^	0.921
Occasionally	49 (6.81)	19.96	20.34 (14.31 ~ 27.6)		
Sometimes	5 (0.69)	23.77	22.97 (14.28 ~ 44.15)		
Often	5 (0.69)	21.6	20.62 (17.42 ~ 27.63)		
Frequency of use of air purifiers^d^					
None	698 (96.94)	20.47	20.03 (15.16 ~ 26.53)	0.65^b^	0.885
Occasionally	16 (2.22)	19.75	18.37 (13.72 ~ 26.68)		
Sometimes	2 (0.28)	22.86	25.04 (14.83 ~ 25.04)		
Often	4 (0.56)	18.57	17.31 (13.49 ~ 28.38)		
Frequency of toilet cleaner use^d^					
None	286 (39.72)	21.23	20.69 (15.24 ~ 28.43)	5.46^b^	1.141
Occasionally	248 (34.44)	19.74	19.16 (14.95 ~ 24.29)		
Sometimes	119 (16.53)	20.78	19.67 (16.06 ~ 26.99)		
Often	67 (9.31)	19.32	18.14 (14.72 ~ 24.46)		
Frequency of hood use^d^					
None	326 (45.28)	21.28	20.63 (15.72 ~ 27.76)	11.88^b^	0.008
Occasionally	230 (31.94)	20.39	20.07 (16.15 ~ 25.85)		
Sometimes	105 (14.58)	19.43	18.47 (13.95 ~ 24.18)		
Often	59 (8.19)	18.18	16.37 (13.52 ~ 22.97)		
Renovation time					
Not renovated	365 (50.69)	20.29	19.47 (14.81 ~ 26.85)	1.45^b^	0.695
< 2 years	55 (7.64)	19.51	19.67 (14.95 ~ 23.86)		
2 ~ 5 years	106 (14.72)	20.88	20.24 (16.32 ~ 25.45)		
> 5 years	194 (26.94)	20.78	20.35 (16.21 ~ 25.97)		
Furniture replacement time					
Not replaced	442 (61.39)	20.56	19.73 (15.16 ~ 27.24)	1.42^b^	0.702
< 2 years	77 (10.69)	19.74	18.29 (14.43 ~ 25.61)		
2 ~ 5 years	100 (13.89)	20	20.14 (16.36 ~ 24.43)		
> 5 years	101 (14.03)	20.95	20.81 (15.52 ~ 26.89)		
Type of housing					
Simple Bungalow	18 (2.5)	23.85	24.52 (19.58 ~ 29.89)	15.90^b^	0.003
Brick Bungalow	27 (3.75)	19.77	20.63 (14.53 ~ 25.48)		
Simple House	349 (48.47)	21.37	20.92 (16.03 ~ 27.76)		
Commercial house	323 (44.86)	19.38	17.98 (14.63 ~ 24.39)		
Villa	3 (0.42)	20.49	20.34 (16.4 ~ 20.34)		
Actual use area of housing (m^2^)					
≤100	319 (44.31)	20.06	19.09 (14.82 ~ 25.83)	3.32^b^	0.190
100–200	301 (41.81)	20.43	20.29 (15.15 ~ 26.12)		
>200	100 (13.89)	21.82	21.5 (16.07 ~ 28.13)		
Kitchen Type					
Closed	473 (65.69)	20.55	20.36 (15.26 ~ 26.29)	−0.67^a^	0.505
Open	247 (34.31)	20.25	19.09 (14.96 ~ 27.03)		
Kitchen ventilation					
Range hoods	15 (2.08)	21.46	23.66 (12.39 ~ 27.73)	4.45^b^	0.217
Exhaust fan	384 (53.33)	20.2	19.46 (15.14 ~ 25.72)		
No measures taken	197 (27.36)	19.98	19.67 (14.83 ~ 26.49)		
Natural window ventilation	124 (17.22)	21.92	20.69 (16.5 ~ 28.29)		
First domestic fuel for cooking					
Firewood/charcoal/wood/animal manure	92 (12.78)	22.33	21.87 (17.02 ~ 28.27)	9.01^b^	0.061
Coal	4 (0.56)	29.25	27.71 (15.91 ~ 63.46)		
Gas/Liquefied Petroleum/Natural Gas/Biogas	595 (82.64)	20.19	19.48 (14.95 ~ 25.99)		
Solar/Electricity	17 (2.36)	20.36	23.04 (14.11 ~ 26.86)		
None	12 (1.67)	17.36	17.14 (12.92 ~ 20.87)		
Frequency of cooking^d^					
None	53 (7.36)	19.11	19.2 (15.15 ~ 23.37)	8.01^b^	0.046
Occasionally	42 (5.83)	17.48	16.57 (13.43 ~ 22.33)		
Sometimes	107 (14.86)	20.37	19.48 (14.68 ~ 26.89)		
Often	518 (71.94)	20.87	20.2 (15.63 ~ 27.36)		
Main heating method in winter					
No	113 (15.69)	21.68	21.37 (15.23 ~ 27.76)	9.10^b^	0.105
Centralized heating	7 (0.97)	19.54	19.48 (17.42 ~ 23.08)		
Gas	5 (0.69)	18.15	19.79 (11.26 ~ 31.01)		
Coal-fired	5 (0.69)	19.74	18.14 (13.79 ~ 31.05)		
Electric heating	516 (71.67)	19.94	19.05 (14.83 ~ 25.72)		
Other	74 (10.28)	22.65	21.81 (17.16 ~ 28.69)		
Frequency of indoor ventilation in spring					
No window	3 (0.42)	18.65	20.36 (14.53 ~ 20.36)	1.45^b^	0.695
1–3 times/week	28 (3.89)	20.2	21.49 (16.95 ~ 25.28)		
3–5 times/week	36 (5)	18.71	19.39 (13.75 ~ 25.54)		
>5 times/week	653 (90.69)	20.57	19.97 (15.13 ~ 27.05)		
Frequency of indoor ventilation in summer					
Without opening windows	1 (0.14)	18.93	18.93 (18.93 ~ 18.93)	0.60^b^	0.897
1–3 times/week	12 (1.67)	21.48	23.28 (16.62 ~ 26.48)		
3–5 times/week	21 (2.92)	19.81	22.06 (15.77 ~ 24.75)		
>5 times/week	686 (95.28)	20.45	19.91 (15.04 ~ 26.72)		
Frequency of indoor ventilation in fall					
No windows open	4 (0.56)	16.89	15.96 (11 ~ 29.07)	2.46^b^	0.483
1–3 times/week	37 (5.14)	18.32	19.19 (13.96 ~ 23.71)		
3–5 times/week	52 (7.22)	20.26	21.58 (15.88 ~ 26.34)		
>5 times/week	627 (87.08)	20.62	19.84 (15.13 ~ 27.03)		
Frequency of indoor ventilation in winter					
No windows open	32 (4.44)	19.95	20.78 (14.65 ~ 26.45)	2.45^b^	0.484
1–3 times/week	142 (19.72)	20.6	21.16 (14.56 ~ 27.33)		
3–5 times/week	91 (12.64)	21.56	20.8 (16.91 ~ 25.03)		
>5 times/week	455 (63.19)	20.22	19.21 (14.95 ~ 26.2)		

^aStatistics are Z-values; bstatistics are H-values; d“Occasionally” refers to a frequency of use of not more than once a week; “Sometimes” refers to a frequency of use of at least once a week but not more than once a day; “Often” refers to a frequency of use of once a day or more.^

### 3.4 Multiple linear regression analysis of factors influencing blood lead

Blood lead concentration was approximately normally distributed after log-transformation, and by stepwise linear regression, gender (*β* = 0.078, *p* < 0.001), age groups (*β* = 0.022, *p* < 0.001), annual household income (>100 thousand yuan) (*β* = −0.042, *p* < 0.05), smoking (*β* = 0.075, *p* < 0.001), hypertension (*β* = 0.092, *p* < 0.05), and frequency of hood use (*β* = −0.014, *p* < 0.05) still had significant effects on blood lead concentration (Table 4).

**Table 4:** Multiple linear regression results of blood lead levels and related factors in permanent residents of Jiangxi Province.

Variables	*β*	*β* standard error	*t*	*p*
Gender ^e^	0.078	0.014	5.79	<0.001
Age group ^f^	0.022	0.004	5.31	<0.001
Annual household income (>100 thousand yuan) ^g^	−0.042	0.015	−2.74	0.006
Smoking ^h^	0.075	0.020	3.64	<0.001
Hypertension ^h^	0.092	0.027	3.38	0.001
Frequency of hood use^i^	−0.014	0.007	−2.09	0.037

Variable assignment: ^e^0 = female ~ 1 = male; ^f^1 = 3 ~ 5 years old ~ 2 = 6 ~ 11 years old ~ 3 = 12 ~ 18 years old ~ 4 = 19 ~ 39 years old ~ 5 = 40 ~ 59 years old ~ 6 = 60 ~ 79 years old; ^g^0 = else ~ 1= > 100 thousand yuan; ^h^0 = no ~ 1 = yes; ^i^1 = None ~ 2 = Occasionally ~ 3 = Sometimes ~ 4 = Often.

### 3.5 Effects of blood lead levels on routine hematological and biochemical indices

The data of routine hematological and biochemical indexes of the surveyed subjects were skewed, and their median, P_25_ and P_75_ distributions are shown in Table 5. We classified the data of routine hematological and biochemical indexes into low, medium, and high levels according to P_25_ and P_75_, and analyzed the effects of blood lead levels on routine hematological and biochemical indexes of the survey respondents after correcting for gender, age, annual household income, smoking, and hypertension. Logistic regression analysis showed that each unit increase in blood lead resulted in a 2.4% (OR = 1.024, 95%CI: 1.001 ~ 1.048), 1.6% (OR = 1.016, 95%CI: 1.002 ~ 1.031), 3.6% (OR = 1.036, 95%CI: 1.000 ~ 1.072), and 2.3% (OR = 1.023, 95%CI: 1.007 ~ 1.039) increased risk of elevated RBC (from low to high levels), PDW, ALP (from low to high levels), and CHO, respectively, while the risks of elevated DBIL and TBIL decreased by 1.7% (OR = 0.983, 95%CI: 0.969 ~ 0.998) (*p* < 0.05) (Table 6).

**Table 5:** Distribution of routine hematological and biochemical indices of permanent residents in Jiangxi Province.

Routine hematological and biochemical indices	M	P_25_	P_75_
RBC (10^12^/L)	4.65	4.25	5.00
Hb (g/L)	140.00	127.25	150.00
MCV (fL)	87.60	82.93	92.40
MCHC (g/L)	341.50	330.00	351.00
PDW (%)	13.35	10.10	15.90
MPV (fL)	9.60	8.70	10.20
AST (IU/L)	24.00	19.35	29.00
ALP (IU/L)	101.00	70.00	191.88
ALT (IU/L)	16.05	12.00	26.00
DBIL (μmol/L)	2.40	1.60	3.70
TBIL (μmol/L)	11.00	7.93	14.50
CHO (mmol/L)	4.33	3.82	5.18

RBC, red blood cell counts; Hb, hemoglobin; MCV, mean corpuscular volume; MCHC, mean corpuscular hemoglobin concentration; PDW, platelet volume distribution width; MPV, mean platelet volume; AST, aspartate aminotransferase; ALP, alkaline phosphatase; ALT, alanine aminotransferase; DBIL, direct bilirubin; TBIL, total bilirubin; CHO, cholesterol; Ref, reference category.

**Table 6:** Association of blood lead levels with routine hematological and biochemical indices in permanent residents of Jiangxi Province.

Dependent variable^**^	*β*	OR (95% CI)	Wald	*p*
RBC (low level) ^j^	Ref	Ref	Ref	Ref
RBC (medium level) ^j^	0.010	1.01 (0.989 ~ 1.030)	0.871	0.351
RBC (high level) ^j^	0.024	1.024 (1.001 ~ 1.048)	4.115	0.043***
Hb (low level) ^j^	Ref	Ref	Ref	Ref
Hb (medium level) ^j^	0.004	1.004 (0.984 ~ 1.024)	0.128	0.721
Hb (high level) ^j^	0.009	1.009 (0.986 ~ 1.033)	0.619	0.431
MCV (low level) ^j^	Ref	Ref	Ref	Ref
MCV (medium level) ^j^	−0.003	0.997 (0.974 ~ 1.021)	0.062	0.803
MCV (high level) ^j^	0.004	1.004 (0.978 ~ 1.031)	0.107	0.743
MCHC ^k^	−0.004	0.996 (0.981 ~ 1.01)	0.326	0.568
PDW ^k^	0.016	1.016 (1.002 ~ 1.031)	4.816	0.028***
MPV ^k^	0.003	1.003 (0.988 ~ 1.018)	0.155	0.694
AST (low level) ^j^	Ref	Ref	Ref	Ref
AST (medium level) ^j^	0.013	1.013 (0.989 ~ 1.038)	1.198	0.274
AST (high level) ^j^	0.024	1.024 (0.998 ~ 1.051)	3.237	0.072
ALP (low level) ^j^	Ref	Ref	Ref	Ref
ALP (medium level) ^j^	0.014	1.014 (0.995 ~ 1.034)	2.200	0.138
ALP (high level) ^j^	0.035	1.036 (1.000 ~ 1.072)	3.916	0.048***
ALT ^k^	0.005	1.005 (0.99 ~ 1.02)	0.458	0.499
DBIL ^k^	−0.017	0.983 (0.969 ~ 0.998)	5.262	0.022***
TBIL ^k^	−0.017	0.983 (0.969 ~ 0.998)	4.901	0.027***
CHO ^k^	0.023	1.023 (1.007 ~ 1.039)	8.044	0.005***

^j^Parallel line test *p* < 0.05 using unordered logistic regression with low level as the reference category. ^k^Ordered logistic regression. RBC, red blood cell counts; Hb, hemoglobin; MCV, mean corpuscular volume; MCHC, mean corpuscular hemoglobin concentration; PDW, platelet volume distribution width; MPV, mean platelet volume; AST, aspartate aminotransferase; ALP, alkaline phosphatase; ALT, alanine aminotransferase; DBIL, direct bilirubin; TBIL, total bilirubin; CHO, cholesterol; Ref, reference category. ***p < 0.05.

## 4 Discussion

The study found that the geometric mean of blood lead concentration in permanent residents of Jiangxi Province was 20.45 μg/L, which was significantly lower than that of Liaoning Province, higher than that of Jilin Province (34) and the national (35). Compared with other countries, the geometric mean of blood lead in our province is higher than that in the United States (36) and Korea (37). The range of blood lead concentration of permanent residents in Jiangxi Province is 7.27 ~ 103.73 μg/L, which is far below the limit value of lead poisoning proposed by the China Health and Wellness Commission (400 μg/L for adults and 100 μg/L for children) and is still at a low concentration level.

In the study, the blood lead levels of males was higher than that of females, which was consistent with Guizhou Province (38). This difference may be due to differences in men’s and women’s jobs and lifestyles, as well as physiological processes and hormone levels. Females are mainly engaged in family activities, and their positions require avoiding lead working environment as much as possible, while males have long outdoor activities and more opportunities for lead occupational exposure, so their blood lead levels are relatively higher. This study found that age was positively correlated with blood lead levels (*p* < 0.001), which may be related to the duration of exposure and metabolic levels in different age groups. Lead tends to accumulate in the human body, and as age increases, the exposure time of the human body to lead increases, while the functions of the body decrease and the metabolic rate also slows down, which makes the accumulation of lead in the body more serious and the internal exposure level increase. It was also found that those with an annual household income of more than 100 thousand yuan had lower blood lead levels (*p* < 0.05), probably because higher-income people have better living environments and pay more attention to the quality of foods than lower-income people, and therefore are less likely to consume lead-containing foods and have less exposure to lead in their living environments. The study showed that the blood lead level was lower in residents with frequent use of hoods, which may be due to the fact that lead is contained in grease fumes, and those with frequent use of hoods inhaled less grease fumes and therefore had a lower blood lead level. There was no statistically significant difference in blood lead exposure between urban and rural residents (*p* > 0.05), which may be related to the industrial layout and the proximity of urban and rural residents’ living standards, as factories tend to build their plants at the urban–rural border to save costs, and as China’s strategy of revitalization of the countryside advances, the living standards of rural residents have significantly improved, and urban and rural residents’ living conditions have become more and more convergent, so that urban and rural residents have similar levels of exposure to lead. Human lead comes from the outside environment, and tobacco smoke is an important source of lead exposure for permanent residents. Tobacco plants can capture lead from soil and air and enrich it in tobacco leaves. The results of this study demonstrated that human blood lead concentrations were higher in the smoking group than in the non-smoking group. The study (39) showed that each gram of cigarette contains about 0.54 μg of lead, and 33–60% of lead is transferred to cigarette smoke during the smoking process and enters the body as aerosols through the respiratory tract, thus the lead level in smokers is usually higher. It is suggested that changing the living habits of the population and advocating smoking cessation are feasible measures to reduce lead exposure. There is a lack of evidence regarding the mechanism of the effect of lead and hypertension, but many cross-sectional studies (40–42) have shown that lead exposure levels are positively associated with hypertension, which is consistent with the results of this study.

Currently, most of the studies on lead and routine hematological and biochemical indices focus on high blood lead internal exposure, and there is a lack of studies on the effects of low blood lead internal exposure on routine hematological and biochemical indices. The low blood lead concentration explored in the study makes up for the shortcomings of this type of study. High concentrations of lead can interfere with the redox reactions and energy metabolism of cells by binding to enzymes containing sulfhydryl groups involved in cellular metabolism, resulting in damage to multiple organ systems such as the hematopoietic system and liver, causing changes such as a decrease in RBC and Hb. The study found that low blood lead concentrations were positively correlated with RBC and CHO and negatively correlated with DBIL and TBIL. This may be related to the toxic excitatory effect (hormesis), which is the stimulating effect of low-concentration blood lead on the organism.

Lead inhibits the activity of heme synthetase, so lead poisoning is often manifested by a decrease in hematocrit, and the body shows signs of anemia. However, the results of this study showed a positive correlation between blood lead exposure and red blood cell count. Although lead blocks the binding of protoporphyrin to iron to form heme and reduces heme synthesis, the low blood lead concentration also leads to a lower degree of hematocrit decline, and the body stimulates the hematopoietic system to produce more erythrocytes through a feedback mechanism, leading to a compensatory increase in the number of erythrocytes to reduce the adverse effects caused by lower hematocrit (43), which results in an increase in the red blood cell count as shown in the assay index. Therefore, the positive correlation between low blood lead exposure and erythrocyte count shown in this study is not contradictory to blood Pb causing anemia. Bilirubin is a metabolite of hemoglobin, and lead may increase bilirubin levels by inducing hemoglobin degradation, while the study found that the risk of elevated DBIL and TBIL levels decreased with increasing blood lead concentration, which is consistent with the study of Ye M et al. (44), probably due to a decrease in bilirubin synthesis caused by the depletion of hemoglobin.

ALP is an enzyme widely distributed in human liver, bones, intestines, kidneys, placenta and other tissues and excreted by liver to bile, which can reflect the function of liver and bile (45). Ali Firoozichahak et al. showed that blood lead levels were positively correlated with ALP levels, which is consistent with my findings (46). On the one hand, lead causes disturbance and disruption of cell membranes (47). Phosphate is known as an intracellular anion and it increases serum ALP levels when cell membranes are damaged or disrupted (48). On the other hand, bones contain large amounts of ALP. Lead replaces bone calcium, leading to structural damage to the bones, thus causing an increase in serum ALP levels (49).

Park et al. (25) found that blood lead levels were positively correlated with total cholesterol levels, which is consistent with the findings of the study, and it may be that elevated blood lead levels induce lipid peroxidation (50), which increases cholesterol through lipid peroxidation. PDW indicates the size distribution of platelets produced by megakaryocytes and is an important marker of platelet activation. Kooshki et al. (51) found a positive correlation between lead exposure and platelet distribution width, which is consistent with the results of the study, suggesting that lead may cause inflammatory responses and altered platelet morphology in the body.

In conclusion, this study found that there is a certain correlation between blood lead concentration and blood routine hematological and biochemical indexes in permanent residents, suggesting that low blood lead concentration may be related to the number of red blood cells, platelet morphology, as well as liver functions. The mechanism of its effect still needs to be studied in depth, and the threshold value of blood lead concentration that has a damaging effect on the hematological system and liver function needs to be further determined. In this study, the effect of low blood lead concentration on routine hematological and biochemical indexes of permanent residents was found, which provides a scientific basis for early identification, prevention, and control of potential health damage from lead and reduction of its health risk. However, our study is a prospective study, which can only provide clues to investigate the association between blood lead and routine blood and biochemical indicators, but cannot determine the causal relationship between them. Therefore, we need to conduct further prospective studies to investigate the causal relationship between blood lead and routine blood and biochemical indicators. In addition, since blood lead is an important indicator of recent lead exposure, but lead tends to accumulate in the body over a long period of time, our future studies could further measure lead in urine and bone, and explore the effects of lead exposure in urine and bone on the health of the population.

## Data Availability

The original contributions presented in the study are included in the article/supplementary materials, further inquiries can be directed to the corresponding author.
